# Metagenomic data of the bacterial community in coastal Gulf of Mexico sediment microcosms following exposure to Macondo oil (MC252)

**DOI:** 10.1016/j.dib.2015.11.040

**Published:** 2015-11-26

**Authors:** Hyunmin Koo, Asim K. Bej

**Affiliations:** Department of Biology, University of Alabama at Birmingham, Birmingham, AL, USA

## Abstract

The data in this article includes the sequences of bacterial 16S rRNA gene from metagenome of Macondo oil (MC252)-treated and non-oil-treated sediment microcosms, collected from coastal Gulf of Mexico and Bayou La Batre, USA. Metacommunity DNA was PCR amplified with 341F and 907R oligonucleotide primers, targeting V3–V5 regions of the 16S rRNA gene. Data were generated by using bacterial tag-encoded FLX-amplicon pyrosequencing (bTEFAP) methodology and then processed using bioinformatics tools such as QIIME. The data information is deposited to NCBI׳s BioProject and BioSample and raw sequence files are available via NCBI׳s Sequence Read Archive (SRA) database.

**Specifications Table**

**Table:** 

Subject area	Biology, Microbial ecology, Biodiversity
More specific subject area	Metagenomics
Type of data	Table
How data was acquired	Pyrosequencing was conducted on a Roche 454 FLX instrument using the Roche Titanium reagents, following the procedures developed at Research and Testing Laboratories (RTL) (Lubbock, TX, USA) (www.researchandtesting.com)
Data format	Raw data sff file
Experimental factors	V3–V5 regions of bacterial 16S rRNA gene were PCR amplified using 341F and 907R oligonucleotide primers.
Experimental features	The sediment samples were collected from 1) Bayou La Batre, AL, USA; 2) U.S. Gulf of Mexico from three different locations (Dauphin Island, Petit Bois Island, and Perdido Pass) and then metacommunity DNA was extracted from non-oil-treated and oil (MC252)-treated sediment samples at various time points for pyrosequencing.
Data source location	1) Bayou La Batre, AL, USA (30°22.7140′N, 88°18.2300ʹW); 2) Dauphin Island (30°14ʹ54″N, 88°4ʹ24″W), Petit Bois Island (30°12ʹ47″N, 88°30ʹ0″W), and Perdido Pass (30°16ʹ45″N, 87°33ʹ22″W), on the Gulf of Mexico, AL, USA.
Data accessibility	The accession numbers for all sequence data in this paper have been listed in Table 1, and are publicly available at NCBI.

**Value of the data**

• This data information provides changes in the microbial community structure and species composition following treatment with MC252.
• Data is applicable for comparative studies related to oil spill events that have occurred in similar or different locations in the Gulf of Mexico or other ecosystems.
• Accessibility of raw sequence data allows researchers to perform new analyses based on their own research purposes with new bioinformatics tools.

## 1 Data

All raw sequence data described in this paper are available through NCBI׳s BioProject, BioSample and SRA database. Accession numbers of BioProject, BioSample and SRA are elaborated in Table 1. The two sets of nextgen sequence data represent the microbial communities from the MC252-treated and non-oil-treated sediment samples in a laboratory microcosm experiment. The sediments were collected from 1) Bayou La Batre, AL, USA; and 2) Dauphin Island, Petit Bois Island, and Perdido Pass, AL, USA.

## 2 Experimental design, materials and methods

### 2.1 Sample collection and preparation

The sediment and seawater samples were collected from Bayou La Batre, AL in March, 2011 using acid-washed plastic containers. The samples were collected from a single location and placed in a 5-gallon acid-washed plastic bucket. Then, the top 15–30 cm of sediments were sampled, which were thoroughly mixed by stirring and then used for the microcosm setup. To confirm the initial level of oil present in the sediment samples, total petroleum hydrocarbons and total organic carbon (TOC) were revealed by GC–MS using standard methods [1], [2], [3]. For each microcosm setup, 200 g (dry weight) sediment and 173.06 g autoclaved (121 °C for 15 min at 15 lb/sq inch pressure) seawater were mixed and placed in a 500 mL glass jar. Then, duplicate sediment samples were subjected to MC252-treatment (500 ppm) for 14 days and 21 days at room temperature (20±1 °C) (Table 1) [4]. Non-oil-treated control samples (0 h) were maintained throughout the experimentation.

The sediment samples mixed with seawater along the coast of Dauphin Island, Petit Bois Island, and Perdido Pass, were collected in June, 2011 in sterile-cap tubes using a multicorer from the upper 5 cm of sediment surface. Samples were stored at 4 °C in sterile capped tubes. Duplicate sediment samples (each 50 g dry weight) were mixed with 200 ml autoclaved seawater in 500 ml glass jars. Then, the non-oil-treated control (0 h) and oil-treated samples were incubated at room temperature (20±1 °C) for 7 days and 30 days (Table 1) [4]. Since the aim of the study was to monitor a short-term effect of the MC252 oil in sediment microbial communities, we have used the treatment time and scheme described previously by Cappello et al [5].

### 2.2 DNA extraction

All sediment samples (1 g each in triplicate) from Bayou La Batre and Gulf of Mexico were subjected to metacommunity DNA extraction by using the MoBIO PowerSoil® DNA Isolation Kit (MoBio Laboratories Inc., CA; www.mobio.com; cat # 12888-100). The quality and concentration of the extracted DNA samples were measured by using a Lambda 2 spectrophotometer (Perkin Elmer, Norwalk, Conn.) followed by agarose gel electrophoresis in Tris-Acetate-EDTA (TAE, pH 7.5) buffer [6].

### 2.3 Sequencing

After confirming the purity and the concentration of the DNA, triplicate samples from each oil-treated and non-oil-treated sediment samples were pooled, and 100 ng of DNA was used by the Research and Testing Laboratories (RTL) (Lubbock, Texas) for bacterial tag-encoded FLX-amplicon pyrosequencing (bTEFAP) [7]. The pyrosequencing was conducted using 341F (5′CCT ACG GGA GGC AGC AG 3′) [8] and 907R (5′CCG TCA ATT CMT TTG AGT TT 3′) [9] oligonucleotide primers targeting the V3–V5 regions of the bacterial 16S rRNA gene [10]. Then, initial generation of the sequencing library was conducted by one-step PCR using the HotStarTaq™ Plus Master Mix Kit (Qiagen, Valencia, CA) and 341F and 907R primers. The pyrosequencing was conducted on a Roche 454® FLX instrument using the Titanium reagents and procedures developed at RTL (Lubbock, TX).

### 2.4 Data analysis

A total of 12 sff files were generated by pyrosequencing and submitted to the NCBI׳s BioProject, BioSample, and SRA with accession numbers listed in Table 1. These sff files can be converted to FASTA- and QUAL-formatted files by using “process_sff.py” command in QIIME (ver 1.8.0). After converting the sff files, a Mapping file, including Sample ID, BarcodeSequence, and LinkerPrimerSequence information, was created for the analyses. After creating the mapping file, formatting requirements in this file were checked by using “validate_mapping_file.py” in QIIME. Then, these three files (FASTA, QUAL, and Mapping) were used for different analyses by using QIIME as described by Koo et al. [11], [12]. All sff files used in this study can be downloaded publicly from NCBI׳s SRA.

## Conflict of interest

The authors declare no conflict of interest associated with this manuscript.

## Figures and Tables

**Table 1:** Samples treatment, data descriptions, BioProject, BioSample, and SRA accession numbers assigned to this data.

Oil treatment time point	USA: Alabama			USA: Gulf of Mexico								
Bayou La Batre (30°22.7140ʹN, 88°18.2300ʹW)			Dauphin Island (30°14ʹ54″N, 88°4ʹ24″W)			Petit Bois Island (30°12ʹ47″N, 88°30ʹ0″W)			Perdido Pass (30°16ʹ45″N, 87°33ʹ22″W)		
	0 h	14 days	21 days	0 h	7 days	30 days	0 h	7 days	30 days	0 h	7 days	30 days

Data description	Non-oil-treated control (0 h)	14-day oil-treated	21-day oil-treated	Non-oil-treated control (0 h)	7-day oil-treated	30-day oil-treated	Non-oil-treated control (0 h)	7-day oil-treated	30-day oil-treated	Non-oil-treated control (0 h)	7-day oil-treated	30-day oil-treated
BioProject No.	PRJNA294625			PRJNA244781								
BioSample No.	SAMN04027582	SAMN04027583	SAMN04027584	SAMN02728982	SAMN02728983	SAMN04028927	SAMN02728987	SAMN02728988	SAMN02728989	SAMN02728984	SAMN02728985	SAMN04028928
SRA No.	SRX1179581	SRX1179585	SRX1179587	SRX523350	SRX523351	SRX1182496	SRX523357	SRX523358	SRX523359	SRX523360	SRX523361	SRX1182007
